# Serum S100A8 as a potential biomarker for diagnosis of antiphospholipid syndrome and risk stratification among aPL carriers

**DOI:** 10.1136/lupus-2025-001873

**Published:** 2026-02-09

**Authors:** Liang Luo, Yuebing Wang, Wenhua Zhu, Xiangjun Liu, Lei Zhu, Ru Li, Chun Li

**Affiliations:** 1 Department of Rheumatology and Immunology, Peking University People’s Hospital, Beijing, China; 2 Chinese Medicine, The People’s Hospital of Yubei District of Chongqing City, Chongqing, China; 3 School of Integrated Chinese and Western Medicine, Hunan University of Chinese Medicine, Changsha, China; 4 Department of Rheumatology and Immunology, Peking University People’s Hospital Qingdao Hospital, Qingdao, China; 5 Department of Clinical Laboratory, The Second People’s Hospital of Nantong, Nantong, China

**Keywords:** Antiphospholipid Syndrome, Risk Factors, Autoimmune Diseases

## Abstract

**Background:**

Current diagnosis of antiphospholipid syndrome (APS) relies on antiphospholipid antibodies (aPL) testing, but false-positive aPL results and asymptomatic aPL carriers pose significant clinical challenges. The importance of S100A8 in thrombosis has been demonstrated, yet its potential role in APS has received little attention. This study aimed to assess serum S100A8 for APS diagnosis and risk stratification among aPL carriers.

**Methods:**

Serum S100A8 levels were measured by ELISA in healthy controls (HCs), aPL carriers without manifestation and patients with APS. Receiver operating characteristic curves were used to evaluate the diagnostic performance of APS. Logistic regression was performed to identify independent variables associated with obstetric morbidity among female aPL carriers.

**Results:**

The study enrolled 120 HCs, 57 aPL carriers and 114 patients with APS. Serum S100A8 levels were significantly higher in aPL carriers (median 44.3 (IQR 35.6–75.4) ng/mL, p<0.001) and patients with APS (52.8 (37.2–79.2) ng/mL, p<0.001) compared with HCs (25 (21.6–31.1) ng/mL). S100A8 showed good diagnostic accuracy for APS (area under the curve (AUC)=0.854, 95% CI 0.803 to 0.907, p<0.001), with similar performance for thrombotic APS (AUC=0.819, 95% CI 0.747 to 0.891, p<0.001) and obstetric APS (AUC=0.874, 95% CI 0.821 to 0.926, p<0.001). Multivariate logistic regression revealed that S100A8 positivity was independently associated with increased obstetric APS risk among aPL carriers (OR 3.335, 95% CI 1.010 to 11.012, p=0.048).

**Conclusion:**

S100A8 may serve as a complementary biomarker for the diagnosis and risk stratification of APS, especially in female aPL carriers at risk of obstetric morbidity. These findings support further investigation into its clinical and mechanistic role in APS pathogenesis.

**WHAT IS ALREADY KNOWN ON THIS TOPIC**

- Diagnosis and risk assessment in antiphospholipid syndrome (APS) are limited by false-positive antiphospholipid antibodies (aPL) results and difficulty in evaluating asymptomatic aPL carriers.
- Although S100A8 is implicated in inflammation-related thrombosis, its role in APS remains insufficiently defined.

**WHAT THIS STUDY ADDS**

- Serum S100A8 levels were significantly higher in asymptomatic aPL carriers and patients with APS.
- S100A8 showed good diagnostic accuracy for APS, comparable accuracy in thrombotic and obstetric APS subsets.
- S100A8 positivity was independently associated with increased obstetric APS risk among aPL carriers.

**HOW THIS STUDY MIGHT AFFECT RESEARCH, PRACTICE OR POLICY**

- S100A8 may serve as a complementary biomarker for the diagnosis and risk stratification of APS, especially in female aPL carriers at risk of obstetric morbidity.

## Introduction

 Antiphospholipid syndrome (APS) is a systemic autoimmune disorder defined by the occurrence of thrombotic events and/or obstetric morbidity in the presence of persistent antiphospholipid antibodies (aPL), including lupus anticoagulant (LA), anticardiolipin antibodies (aCL) and anti-β2 glycoprotein I antibodies (aβ2GPI).^1^ Epidemiological data indicate that APS affects 1–2 individuals per 100 000 annually, with a prevalence of up to 50 per 100 000 and a rising trend.^2 3^ APS primarily affects young individuals, and severe thrombotic events can be life-threatening, with mortality reaching 50% in cases of catastrophic APS.^4^ Although current classification criteria rely heavily on aPL detection, laboratory assays are sometimes limited by false positives and variable clinical correlation.^5^ Importantly, clinical manifestations remain central to diagnosis, yet the management of persistently aPL carriers without definite APS features is still difficult due to the absence of reliable biomarkers for risk stratification.

S100A8 is a calcium-binding protein highly expressed in neutrophil cytoplasm, where it forms a heterodimer with S100A9 known as calprotectin. S100A8/A9 is stored in the cytosolic fraction and is released predominantly during the formation of neutrophil extracellular traps (NETs).^6^ Recent studies have highlighted the role of neutrophils and NETs, which are prothrombotic and proinflammatory networks of DNA and proteins, in the pathophysiology of APS.^7 8^ Elevated levels of circulating NET remnants have been observed in patients with APS,^9^ and aPL can trigger NET release by activating neutrophils.^10^ Once released, calprotectin promotes inflammation and thrombosis by engaging pattern recognition receptors, such as Toll-like receptor 4 (TLR4) and the receptor for advanced glycation end-products (RAGE), contributing to endothelial activation and leucocyte recruitment.^11 12^ Elevated S100A8 levels have also been reported in thrombosis.^13^ However, its clinical relevance in APS, particularly in obstetric APS (OAPS), remains poorly defined. This study aimed to investigate serum S100A8 levels among healthy controls (HCs), aPL carriers without clinical manifestations and patients with APS. Additionally, we assessed the diagnostic performance of S100A8 in identifying APS and its clinical subtypes, including thrombotic APS (TAPS) and OAPS, and explored its potential association with morbidity in aPL carriers.

## Materials and methods

### Study design and participants

**Figure 1:**
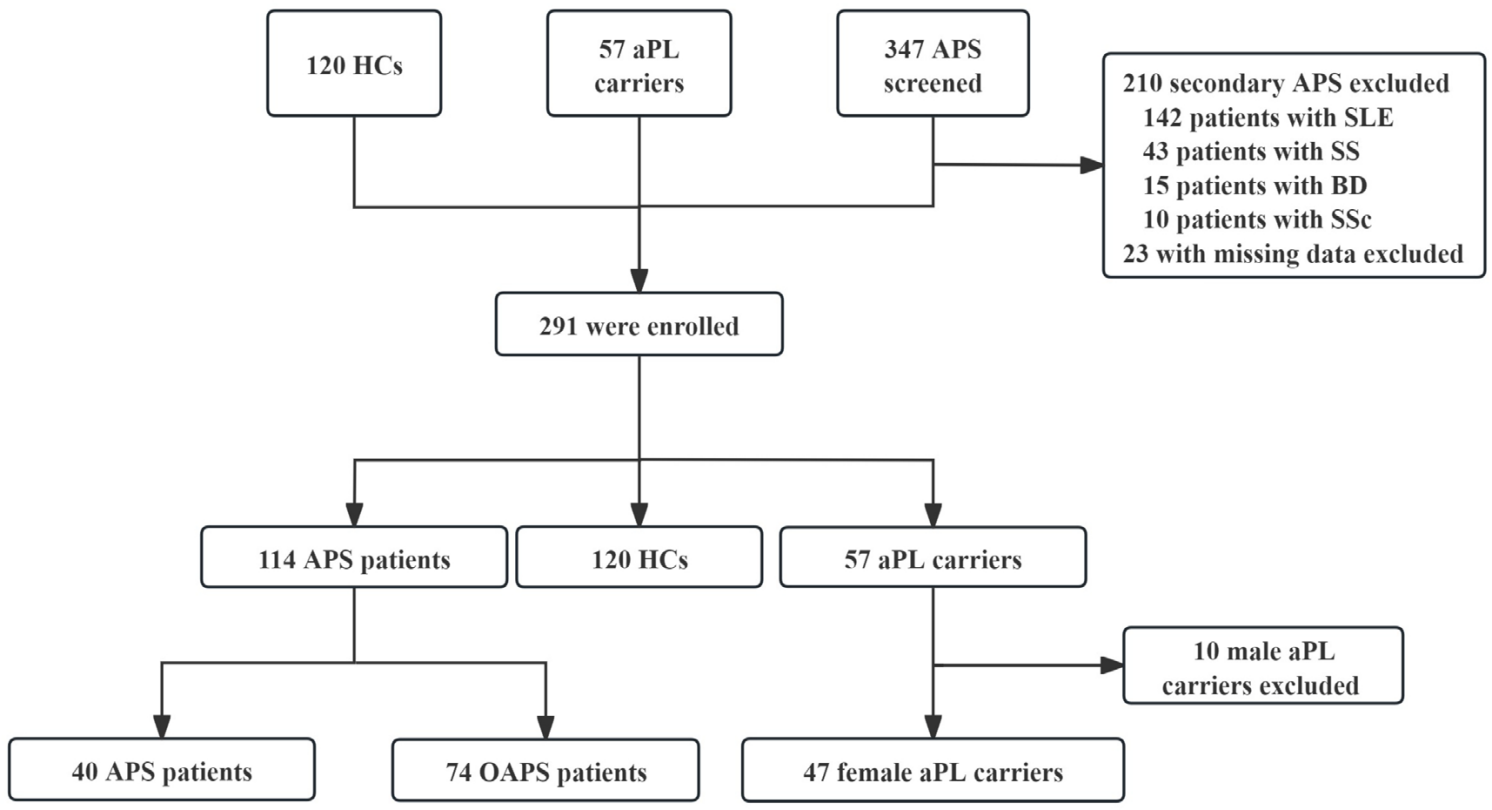
Flowchart of the study. aPL carriers, antiphospholipid antibody positive without clinical manifestations; APS, antiphospholipid syndrome; BD, Behçet's disease; HCs, healthy controls; OAPS, obstetric APS; SS, Sjögren syndrome; SSc, systemic sclerosis; TAPS, thrombotic APS.

This was a cross-sectional study conducted at Peking University People’s Hospital between June 2016 and August 2024. A total of 524 individuals were initially screened. After the exclusion of ineligible participants, 291 participants were enrolled, including HCs, aPL carriers and primary APS. The flowchart of the study is shown in figure 1.

All patients with APS fulfilled the 2006 Sydney and 2023 American College of Rheumatology/European Alliance of Associations for Rheumatology classification criteria of APS.^1 14^ Patients with APS were further classified into TAPS and OAPS based on their clinical manifestations. aPL carriers were defined as those who persistently tested positive for aPL (including LA, aCL or aβ2GPI) at least two times 12 weeks apart but without any clinical manifestations. HCs were age- and sex-matched volunteers with no history of autoimmune disease, thrombosis, pregnancy complications or aPL positivity. Individuals with secondary APS due to SLE or other autoimmune disorders, or with missing clinical or laboratory data, were excluded.

### Data collection

Demographics, cardiovascular risk factors, clinical manifestations and laboratory data were collected from the hospital’s electronic medical records at the time of enrolment. Demographics included age and sex. Cardiovascular risk factors comprised smoking, hypertension, hyperlipidaemia, diabetes and coronary heart disease. Clinical manifestations included thrombotic events and obstetric morbidity. Laboratory indicators included haematological abnormalities (leucopenia, anaemia and thrombocytopenia), aPL (aβ2GPI, aCL and LA), inflammatory markers (C reactive protein and erythrocyte sedimentation rate) and complement components. Treatment included low-dose aspirin (LDA), anticoagulants, hydroxychloroquine (HCQ) and immunosuppressants.

### Serum S100A8 testing

**Figure 2:**
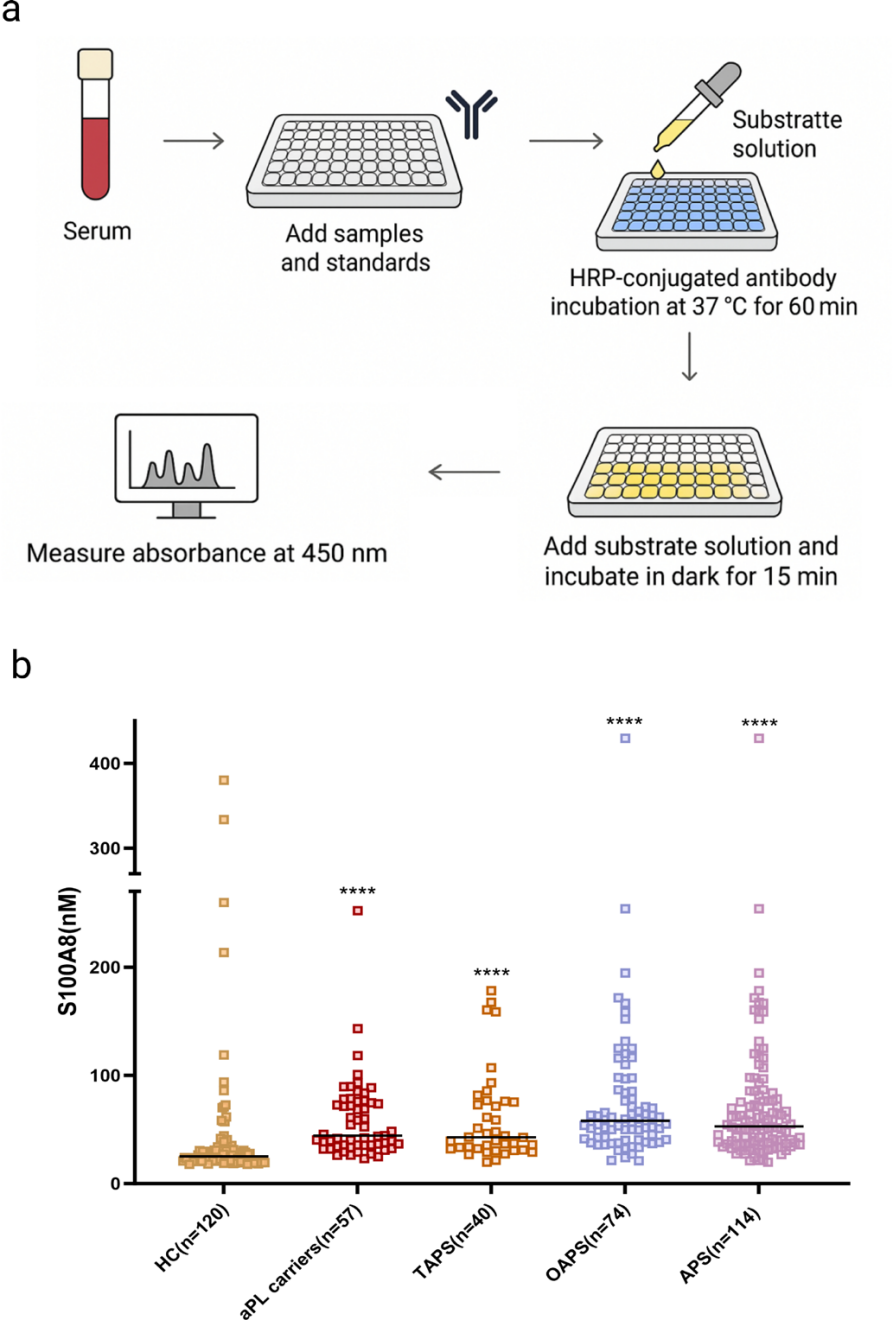
Measurement of serum S100A8 in 291 research subjects. (**a**) Schematic illustration of the S100A8 ELISA. (**b**) Differences in S100A8 levels among HCs, aPL carriers and patients with APS. ****p<0.001 compared with HCs. aPL carriers, antiphospholipid antibody positive without clinical manifestations; APS, antiphospholipid syndrome; HCs, healthy controls; OAPS, obstetric APS; TAPS, thrombotic APS.

Serum S100A8 levels were measured using a commercial human ELISA kit (CHEJETER, China; Catalogue No. CJT12029; 96T format) following the manufacturer’s instructions. The ELISA kit specifically detects free S100A8 monomers. This assay exhibits negligible cross-reactivity with the S100A8/A9 heterodimer (calprotectin) due to conformational masking of target epitopes in the heterocomplex. Samples and standards were added to pre-equilibrated wells, followed by horseradish peroxidase-conjugated antibody incubation at 37 °C for 60 min. After washing, substrate solutions were added and incubated in the dark for 15 min. The reaction was stopped, and absorbance was measured at 450 nm (figure 2a). All samples were tested in duplicate, and mean values were used for analysis.

### Statistical analysis

Continuous variables were tested for normality using the single-sample Kolmogorov-Smirnov test. Normally distributed data are presented as mean±SD and compared using the Student’s t-test. Non-normally distributed data are expressed as median with IQR and compared using the Mann-Whitney U test or Kruskal-Wallis test, as appropriate. Categorical variables are presented as frequencies and percentages and were compared using the χ^2^ test or Fisher’s exact test. The Bonferroni correction was applied for multiple comparisons where appropriate. As the proportion of missing data was <5% and the missingness was random, we did not apply data imputation.

Receiver operating characteristic (ROC) curves were performed to evaluate the diagnostic performance of serum S100A8 in identifying APS and its clinical subtypes. The area under the curve (AUC), optimal cut-off, sensitivity and specificity were calculated. Binary logistic regression analysis was performed to identify independent variables associated with obstetric morbidity among aPL carriers. Variables with p<0.05 in univariate analysis were entered into multivariate models (adjusted for aβ2GPI and LA). All statistical analyses were performed using SPSS V.26.0 (SPSS, Chicago, Illinois, USA) and GraphPad Prism V.10.1.2.

## Results

### Demographic and laboratory characteristics

A total of 120 HCs, 57 aPL carriers and 114 patients with APS, including 40 with TAPS and 74 with OAPS, were enrolled in this study. The baseline characteristics of HCs, aPL carriers and patients with APS are summarised in table 1. Compared with aPL carriers, TAPS patients were older (53.3±16.6 vs 40.3±12.1 years, p=0.005), while OAPS patients tended to be younger (36.1±6.0 vs 40.3±12.1 years, p=0.018). Hypertension was more common in patients with APS than in aPL carriers (20.2% vs 5.3%, p=0.010), especially those with TAPS (47.5% vs 5.3%, p<0.001). A higher rate of coronary heart disease (17.5% vs 1.8%, p=0.008), anticoagulant use (50.0% vs 28.1%, p=0.047) and a lower rate of LDA use (15.0% vs 52.6%, p<0.001) were observed in patients with TAPS compared with aPL carriers. In addition, aPL carriers had higher positivity rates of aCL compared with patients with OAPS (43.9% vs 27.0%, p=0.044). aPL carriers had a lower rate of ANA positivity than patients with APS (19.3% vs 39.5%, p=0.026). After Bonferroni correction (p<0.002), the high prevalence of hypertension and use of LDA in patients with TAPS remained significant.

**Table 1:** Characteristics of the study population

Variables	HCs (n=120)	aPL carriers (n=57)	Patients with APS (n=114)	Patients with TAPS (n=40)	Patients with OAPS (n=74)	P value*	P value†	P value‡
Age, years (x̄±s)	42.4±12.7	40.3±12.1	42.1±13.6	53.3±16.6	36.1±6.0	0.394	0.005	0.018
Female, n (%)	75 (62.5)	47 (82.5)	103 (90.4)	29 (72.5)	74 (100.0)	0.138	0.241	0.001§
Smoking, n (%)	0 (0)	3 (5.3)	8 (7.0)	8 (20.0)	0 (0)	0.912	0.054	0.080
Hypertension, n (%)	0 (0)	3 (5.3)	23 (20.2)	19 (47.5)	4 (5.4)	0.010	<0.001§	1.000
Hyperlipidaemia, n (%)	0 (0)	4 (7.0)	8 (7.0)	7 (63.6)	1 (1.4)	1.000	0.201	0.166
Diabetes, n (%)	0 (0)	4 (7.0)	6 (5.3)	6 (15.0)	0 (0)	0.908	0.203	0.034
Coronary heart disease, n (%)	0 (0)	1 (1.8)	7 (6.1)	7 (17.5)	0 (0)	0.271	0.008	0.435
Thrombosis, n (%)	0 (0)	0 (0)	40 (35.1)	40 (100.0)	0 (0)	–	–	–
Obstetric morbidity, n (%)	0 (0)	0 (0)	74 (64.9)	0 (0)	74 (100.0)	–	–	–
Leucopenia, n (%)	0 (0)	1 (1.9)	6 (5.6)	5 (13.2)	1 (1.4)	0.429	0.092	1.000
Anaemia, n (%)	0 (0)	5 (9.5)	18 (16.7)	11 (28.9)	7 (10.0)	0.234	0.018	0.944
Thrombocytopenia, n (%)	0 (0)	0 (0)	18 (15.8)	13 (32.5)	5 (6.8)	–	–	–
ANA (+), n (%)	0 (0)	11 (19.3)	45 (39.5)	17 (42.5)	28 (37.8)	0.026	0.013	0.021
Anti-β2 glycoprotein I antibodies (+), n (%)	0 (0)	42 (73.7)	85 (74.6)	24 (60.0)	61 (82.4)	0.902	0.155	0.226
Anticardiolipin antibodies (+), n (%)	0 (0)	25 (43.9)	39 (34.4)	19 (47.5)	20 (27.0)	0.219	0.723	0.044
Lupus anticoagulant (+), n (%)	0 (0)	27 (47.4)	47 (41.2)	21 (52.5)	26 (35.1)	0.445	0.619	0.157
Elevated erythrocyte sedimentation rate, n (%)	0 (0)	4 (9.3)	14 (21.9)	9 (31.0)	5 (14.3)	0.088	0.019	0.742
Elevated C reactive protein, n (%)	0 (0)	1 (3.1)	12 (18.5)	3 (10.3)	9 (25.0)	0.055	0.338	0.028
Low complement 3, n (%)	0 (0)	13 (22.8)	17 (18.1)	10 (26.3)	13 (17.6)	0.481	0.696	0.456
Low complement 4, n (%)	0 (0)	9 (18.4)	19 (20.0)	12 (31.6)	7 (12.3)	0.815	0.153	0.383
Treatment, n (%)								
Anticoagulant	–	16 (28.1)	60 (52.6)	20 (50.0)	40 (54.2)	0.004	0.047	0.005
Low-dose aspirin	–	30 (52.6)	55 (48.2)	6 (15.0)	49 (66.2)	0.705	<0.001§	0.163
Hydroxychloroquine	–	42 (73.7)	71 (62.3)	21 (52.5)	50 (67.6)	0.189	0.053	0.571
Immunosuppressant	–	22 (38.6)	32 (28.1)	22 (55.0)	10 (13.5)	0.222	0.164	0.002

Data are presented as mean±SD or number (percentage).

* aPL (+) versus APS.

† aPL (+) versus TAPS.

‡ aPL (+) versus OAPS.

§ Significant differences remained after Bonferroni adjustment. A two-sided Bonferroni adjusted p value of <0.002 (0.05/24) was the threshold for statistical significance.

aPL carriers, antiphospholipid antibody positive without clinical manifestations; APS, antiphospholipid syndrome; HCs, healthy controls; OAPS, obstetric APS; TAPS, thrombotic APS.

Details of manifestations of OAPS phenotypes in patients were shown in online supplemental table 1. Compared with HCs, serum S100A8 levels were elevated in both isolated early pregnancy loss (59.7 (46.3–86.8) ng/mL, p<0.001) and isolated late pregnancy complication patients (61.7 (49.9–98.1) ng/mL, p<0.001). Moreover, S100A8 levels were significantly higher in late complication patients compared with aPL carriers (48.1 (35.1–77.4) ng/mL, p=0.020). There was no significant difference in serum S100A8 levels between patients with isolated early pregnancy loss and isolated late complications (online supplemental figure 1).

### S100A8 levels among the study population

Serum S100A8 levels were significantly elevated in aPL carriers and patients with APS compared with HCs. The median S100A8 concentration in HCs was 25 (IQR 21.6–31.1) ng/mL, which was markedly lower than that in aPL carriers (44.3 (35.6–75.4) ng/mL, p<0.001) and patients with APS (52.8 (37.2–79.2) ng/mL, p<0.001) (figure 2b). The median S100A8 concentration in patients with TAPS and OAPS was 42.8 (32.3–76.0) ng/mL and 58 (40.5–85.7) ng/mL, respectively, which was significantly higher than HCs (figure 2b).

### Diagnostic performance of S100A8

**Figure 3:**
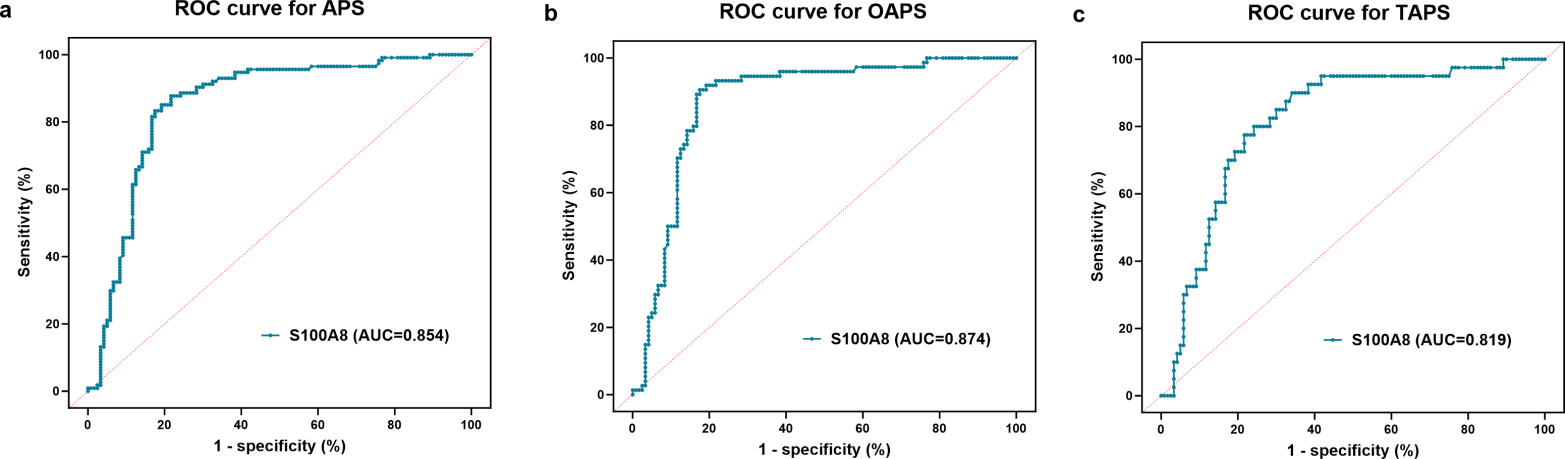
ROC curves of S100A8 for identifying APS and different clinical subtypes. (**a**) ROC curve for overall APS patients; (**b**) ROC curve for TAPS; (**c**) ROC curve for OAPS. The AUC values are shown in each panel. APS, antiphospholipid syndrome; AUC, area under the curve; OAPS, obstetric APS; ROC, receiver operating characteristic; TAPS, thrombotic APS.

The AUC for identifying overall patients with APS was 0.854 (95% CI 0.803 to 0.907), with a sensitivity of 87.7% and a specificity of 78.3% at the optimal cut-off value of 32.19 ng/mL (p<0.001). In subgroup analyses, S100A8 also demonstrated high diagnostic accuracy for both TAPS and OAPS. The AUC was 0.819 (95% CI 0.747 to 0.891) for TAPS, with 80% sensitivity and 75.8% specificity at a cut-off of 31.10 ng/mL (p<0.001). For OAPS, the AUC was 0.874 (95% CI 0.821 to 0.926), with 90.5% sensitivity and 82.5% specificity at a cut-off of 34.02 ng/mL (p<0.001) (figure 3 and online supplemental table 2).

### Subgroup analysis

Baseline demographic and laboratory characteristics of female aPL carriers were shown in online supplemental table 3. After adjusting for aβ2GPI, LA positivity, LDA, HCQ and immunosuppressants, multivariate logistic regression analysis showed S100A8 positivity (OR 3.335, 95% CI 1.010 to 11.012, p=0.048) and ANA positivity (OR=5.439, 95% CI 1.834 to 16.131, p=0.002) were independently associated with an increased risk of OAPS among aPL carriers (table 2). No significant associations were observed between aPL carriers and patients with TAPS.

**Table 2:** Binary logistic regression analysis of predictors of obstetric morbidity among female aPL carriers

Variables	Univariate analysis				Multivariate analysis			
	B	OR	95% CI	P value	B	OR	95% CI	P value
Age, years	−0.042	0.959	0.917 to 1.002	0.064	−0.031	0.970	0.921 to 1.020	0.236
S100A8 (+), n (%)	1.073	2.925	1.044 to 8.197	0.048	1.204	3.335	1.010 to 11.012	0.048
ANA (+), n (%)	0.944	2.570	1.082 to 6.105	0.032	1.694	5.439	1.834 to 16.131	0.002
aβ2GPI (+), n (%)	0.475	1.609	0.662 to 3.909	0.294	0.216	1.241	0.425 to 3.624	0.693
Anticardiolipin antibodies (+), n (%)	−0.693	0.500	0.231 to 1.083	0.079	−0.821	0.440	0.175 to 1.107	0.081
LA (+), n (%)	−0.549	0.577	0.274 to 1.217	0.149	−0.735	0.479	0.184 to 1.248	0.132
Anticoagulant, n (%)	0.920	2.510	1.168 to 5.393	0.018	0.843	2.324	0.861 to 6.275	0.096
LDA, n (%)	0.545	1.725	0.816 to 3.646	0.153	0.500	1.649	0.582 to 4.667	0.346
HCQ, n (%)	−0.336	0.714	0.316 to 1.616	0.419	−0.642	0.526	0.190 to 1.459	0.217
Immunosuppressant, n (%)	−0.895	0.409	0.162 to 1.029	0.058	−0.287	0.750	0.250 to 2.256	0.609

Multivariate analysis was adjusted for aβ2GPI, LA positivity, LDA, HCQ and immunosuppressant.

aβ2GPI, anti-β2-glycoprotein I antibodies; B, regression coefficient; HCQ, hydroxychloroquine; LA, lupus anticoagulant; LDA, low-dose aspirin.

## Discussion

This study showed that serum S100A8 levels were significantly elevated in aPL carriers and patients with APS. Moreover, S100A8 exhibited good diagnostic performance for identifying APS and its subtypes. Multivariate logistic regression analysis revealed that S100A8 positivity was independently associated with an increased risk of obstetric morbidity among female aPL carriers.

The elevated serum S100A8 levels observed in patients with APS may reflect enhanced neutrophil activation and NETs formation, processes implicated in both thrombotic and obstetric manifestations. In TAPS, NETs have been shown to promote endothelial injury and thrombosis through the activation of platelets and the coagulation cascade.^15 16^ However, the most pronounced increase in S100A8 levels was observed in patients with OAPS, suggesting an important role in pregnancy-related complications. aPL can trigger the release of NETs at the maternal–foetal interface, leading to increased reactive oxygen species production and trophoblast apoptosis, ultimately contributing to placental injury in OAPS.^17^ As a major cytosolic component of NETs, S100A8 may engage innate immune receptors such as TLR4 and RAGE on decidual and endothelial cells, promoting inflammation, endothelial dysfunction and trophoblast injury.^18^ These mechanisms may underlie the association between S100A8 and obstetric morbidity, highlighting its potential as a biomarker of placental immune dysregulation in OAPS.

The clinical spectrum of OAPS is heterogeneous, encompassing both recurrent early losses and late pregnancy complications such as severe pre-eclampsia and foetal growth restriction. Notably, the latter is considered more specific to APS pathology.^14 19^ Interestingly, our study found that serum S100A8 levels were similarly elevated in patients with isolated recurrent early losses and those with isolated late pregnancy complications. Both subgroups had significantly higher S100A8 levels compared with HCs. This phenotypic independence suggests that the elevated S100A8 may reflect a common, upstream proinflammatory endothelial environment driven by aPL, serving as a shared risk for various obstetric manifestations.

Although the role of S100A8/A9 has been explored in other autoimmune diseases,^20^,^22^ its involvement in APS, especially in OAPS, remains inadequately characterised. Elevated calprotectin levels have been observed in primary APS patients and in asymptomatic aPL carriers, indicating a potential contribution to disease processes such as endothelial activation and thrombus formation.^23^ While the S100A8/A9 heterodimer (calprotectin) is established as a downstream thrombotic effector that directly activates platelets via epithelial growth factor receptor-dependent pathways to promote coagulation,^24^ free S100A8 functions as an upstream inflammatory alarmin. S100A8 initiates vascular inflammation by activating the endothelial TLR4/mitogen-activated protein kinase axis and amplifying the NOD-, LRR- and pyrin domain-containing protein 3 (NLRP3)/Caspase-1/interleukin-1β pathway,^25 26^ which are potential processes in APS-related endothelial injury and placental dysfunction.^27 28^ Prior evidence suggests that elevated circulating free S100A8 serves as an early diagnostic biomarker in thromboembolic diseases.^29^ Our findings revealed significantly elevated serum S100A8 levels in both patients with APS and aPL carriers compared with HCs, suggesting early immune or vascular activation prior to clinical onset.

Our results showed that S100A8 has good diagnostic potential for APS and its subtypes. The current diagnosis of APS requires both clinical manifestations and persistent positivity for aPL.^14^ However, this approach has notable limitations. Some patients initially test negative for standard aPLs despite clinical features suggestive of APS. In our previous study, the inclusion of non-criteria aPLs significantly enhanced diagnostic accuracy in seronegative APS.^30^ Moreover, aPL levels may fluctuate over time, and their correlation with clinical events is not always consistent.^31^ Therefore, independent of traditional aPL profiles, S100A8 may provide complementary diagnostic value in APS, particularly in cases with ambiguous clinical presentations or borderline serological findings.

This study indicated S100A8 positivity was independently associated with increased OAPS risk among aPL carriers. Given its presumed involvement in neutrophil activation and NETs formation,^6^ S100A8 may contribute to placental immune dysregulation and inflammation, potentially impairing pregnancy outcomes. These findings suggest that S100A8 may serve not only as a biomarker reflecting heightened neutrophil-driven inflammation but also as a potential effector molecule linking innate immune activation to thrombogenic processes in OAPS.^32^ Its upregulation could exacerbate endothelial dysfunction^33^ and promote procoagulant activity, thereby facilitating placental thrombosis and foetal loss. Further mechanistic studies are warranted to elucidate whether targeting the S100A8–NETs axis could mitigate inflammatory and thrombotic cascades in OAPS.

Our study has several limitations. First, the relatively small sample size, especially in subgroup analyses, may reduce the statistical power of the findings. Second, all participants were recruited from a single centre, which may limit the generalisability of the results. Third, the study lacked mechanistic investigations to clarify the role of S100A8 in APS-related immune or inflammatory pathways. Fourth, the retrospective design limited the availability of complete IgG/IgM isotype data for aPLs, precluding a comprehensive cohort-wide analysis of isotype–phenotype associations. Further multicentre studies incorporating basic experimental research are needed to validate and expand upon these findings.

## Conclusion

Serum S100A8 levels were significantly elevated in patients with APS and aPL carriers. S100A8 demonstrated good diagnostic performance and was independently associated with obstetric morbidity. These findings suggest its potential as a complementary biomarker for APS diagnosis and obstetric risk assessment among aPL carriers.

## Supplementary material

10.1136/lupus-2025-001873 online supplemental file 1

10.1136/lupus-2025-001873 online supplemental file 2

## Data Availability

Data are available upon reasonable request.
