# Supplementary figures and images for: Serum S100A8 as a potential biomarker for diagnosis of antiphospholipid syndrome and risk stratification among aPL carriers
Source: Lupus Sci Med. 2026 Feb 9;13(1):e001873. doi: 10.1136/lupus-2025-001873 (PMC12887475; doi:10.1136/lupus-2025-001873)

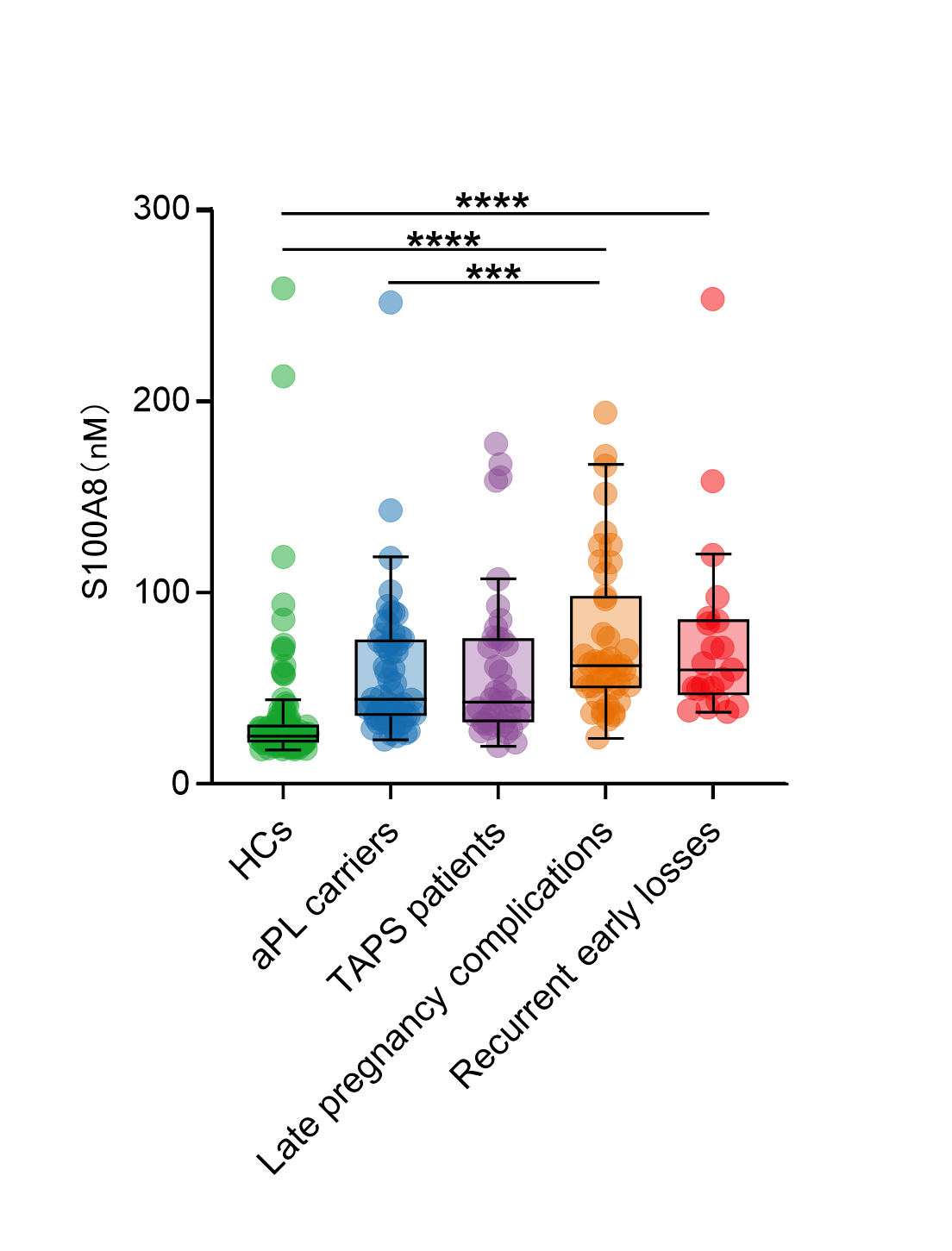

Supplement: online supplemental file 1 [file lupus-13-1-s001.tif]
