# Supplementary material for: Serum S100A8 as a potential biomarker for diagnosis of antiphospholipid syndrome and risk stratification among aPL carriers
Source: Lupus Sci Med. 2026 Feb 9;13(1):e001873. doi: 10.1136/lupus-2025-001873 (PMC12887475; doi:10.1136/lupus-2025-001873)
Supplement: online supplemental file 2 [file lupus-13-1-s002.docx]

## Supplementary Table 1 Pregnancy morbidity in patients with OAPS.

| Pregnancy morbidity, n (%) | OAPS patients  (n = 74) |
| --- | --- |
| Isolated recurrent early losses (≥3 consecutive, <10 weeks fetal loss) | 21 (28.4) |
| Isolated late pregnancy complications | 43 (58.1) |
| ≥10 weeks fetal loss | 23 (31.1) |
| Severe preeclampsia | 12 (16.2) |
| Placental insufficiency (including IUGR) | 9 (12.2) |
| Both recurrent early losses and late pregnancy complications | 10 (13.5) |

APS, antiphospholipid syndrome; OAPS, obstetric APS; IUGR,intrauterine growth restriction.

**Supplementary Table 2** Receiver operating characteristic curve identifying the best threshold of S100A8 for APS, TAPS, and OAPS patients.

| Patients | Cutoff points, nM | 95% CI | AUC | Sensitivity | Specificity | *P* |
| --- | --- | --- | --- | --- | --- | --- |
| APS | 32.19 | 0.803–0.907 | 0.854 | 87.7% | 78.3% | < 0.001 |
| TAPS | 31.10 | 0.747–0.891 | 0.819 | 80.0% | 75.8% | < 0.001 |
| OAPS | 34.02 | 0.821–0.926 | 0.874 | 90.5% | 82.5% | < 0.001 |

APS, antiphospholipid syndrome; TAPS, thrombotic APS; OAPS, obstetric APS; CI, confidence interval; AUC, area under the curve.

**Supplementary Table 3** Characteristics of female aPL carriers.

| Variables | Female aPL carriers  (n=47) |
| --- | --- |
| Age, years (x̄±s) | 39.1 ± 11.2 |
| Smoking, n (%) | 1 (2.1) |
| Hypertension, n (%) | 1 (2.1) |
| Hyperlipidemia, n (%) | 3 (6.4) |
| Diabetes, n (%) | 1 (2.1) |
| Leukopenia, n (%) | 1 (2.4) |
| Anemia, n (%) | 5 (11.9) |
| ANA (+), n (%) | 9 (19.1) |
| aβ2GPI (+), n (%) | 35 (74.5) |
| aCL (+), n (%) | 20 (42.6) |
| LA (+), n (%) | 23 (48.9) |
| Elevated ESR, n (%) | 3 (8.6) |
| Elevated CRP, n (%) | 1 (4.2) |
| Low C3, n (%) | 11 (23.4) |
| Low C4, n (%) | 8 (20.9) |
| Treatment, n (%) |  |
| Anticoagulant | 15 (31.9) |
| LDA | 25 (53.2) |
| HCQ | 35 (74.5) |
| Immunosuppressant | 13 (27.7) |

Data are presented as mean ± SD or number (percentage). aPL (+), antiphospholipid antibody positive without clinical manifestations; OAPS, obstetric antiphospholipid syndrome; CHD, oronary heart disease; ANA, anti-nuclear antibody; aβ2GPI, anti-β2-glycoprotein I antibodies; aCL, anticardiolipin antibodies; LA, lupus anticoagulant; C3, complement 3; C4, complement 4; LDA, low dose aspirin; HCQ, hydroxychloroquine.
